# Videoconferencing Software Options for Telemedicine: A Review for Movement Disorder Neurologists

**DOI:** 10.3389/fneur.2021.745917

**Published:** 2021-10-11

**Authors:** Esther Cubo, Adrian Arnaiz-Rodriguez, Álvar Arnaiz-González, José Francisco Díez-Pastor, Meredith Spindler, Adriana Cardozo, Alvaro Garcia-Bustillo, Zoltan Mari, Bastiaan R. Bloem

**Affiliations:** ^1^Department of Neurology, Hospital Universitario Burgos, Burgos, Spain; ^2^Escuela Politécnica Superior, Universidad de Burgos, Burgos, Spain; ^3^Department of Neurology, University of Pennsylvania, Philadelphia, PA, United States; ^4^Parkinson and Movement Disorders Section, Institute of Neurology, Hospital de Clínicas, Montevideo, Uruguay; ^5^Cleveland Clinic Lou Ruvo Center for Brain Health, Las Vegas, NV, United States; ^6^Department of Neurology, Centre of Expertise for Parkinson & Movement Disorders, Donders Institute for Brain, Cognition and Behaviour, Radboud University Medical Centre, Nijmegen, Netherlands

**Keywords:** telemedicine, movement disorders, Parkinson's disease, videoconference, telehealth

## Abstract

**Background:** The use of telemedicine has increased to address the ongoing healthcare needs of patients with movement disorders.

**Objective:** We aimed to describe the technical and basic security features of the most popular telemedicine videoconferencing software.

**Methods:** We conducted a systematic review of articles/websites about “Telemedicine,” “Cybersecurity,” and “Videoconferencing software.” Technical capabilities and basic security features were determined for each videoconferencing software.

**Results:** Twenty-six videoconferencing software programs were reviewed, 13 (50.0%) were specifically designed for general healthcare, and 6/26 (23.0%) were compliant with European and US regulations. Overall technical and security information were found in 5/26 software (19.2%), including Microsoft Teams, Google Hangout, Coviu, Doxy.me, and Thera platforms.

**Conclusions:** Detailed information about technical capabilities and data security of videoconferencing tools is not easily and openly retrievable. Our data serves as a guide for practitioners seeking to understand what features should be examined when choosing software and what options are available.

## Background

Advances in technology have expanded telemedicine opportunities in medical practice, research, and education. After the declaration of the COVID-19 outbreak as a pandemic, the use of telemedicine has increased to address the ongoing healthcare needs of patients with chronic illnesses, for example, by the introduction of interdisciplinary telehealth services (1–3). Such services have helped reduce the number of in-person clinic visits and thereby minimize human exposures to Coronavirus. In response to the surging needs for remote care, many countries worldwide have expanded laws and regulations to permit greater adoption of telemedicine systems, have provided increased guidance on digital health technologies and cybersecurity expectations, and have expanded reimbursement options (4, 5). Many organizations, including the American Academy of Neurology and the International Parkinson and Movement Disorder Society, have also issued telemedicine guidelines (6, 7).

As demands increased, the pandemic caused a global surge in the use of videoconferencing tools (8). Movement disorders may be considered particularly fitting for distance health/remote visits with videoconferencing, because of the critical importance of observing phenomenology, visual aspects of the exam, speed, presence, distribution, and characteristics of tremor, dyskinesias, etc. In addition, patients with movement disorders are characterized by mobility limitations, and the sparse distribution of movement disorder specialists increasing the difficulty to access (1). Even before telehealth burst into the forefront, movement disorder specialists have been gathering videos of patients for decades at major meetings and weekly video conferences within their group. However, physicians need unbiased and expert guidance in choosing a video conferencing software, including insights into the legal framework, technical capabilities, licenses, patients' access, and costs. Compliance with software data protection requirements is likely to be different worldwide. Examples in data protection regulations include the European Union General Data Protection Regulation (GDPR), which is essential for protecting personal data in Europe. In North America, physicians would look for Health Insurance Portability and Accountability Act (HIPAA) compliant software. Given the increasing offers for videoconferencing in the market, in this article, we describe the technical and basic security standard features of the most popular telemedicine videoconferencing software platforms to inform neurologists interested in developing telemedicine programs. This review is not aimed to provide international or national-based legal information for videoconferencing tools.

## Procedure

For the selection of recent videoconferencing software, we conducted a systematic review of articles published since January 2020 from Medical and Telemedicine Societies, PubMed, and Google using the following keywords: “Telemedicine,” “Cybersecurity,” and “Videoconferencing software.” Only articles and websites in English with detailed information about videoconferencing software characteristics were reviewed. We excluded supplementary applications designed to increase the security to access electronic health medical records or video-based pose estimation of movements with artificial intelligence-based analysis. The following characteristics were determined for each videoconferencing software: chat capability (ability to send/receive text messages), call capability (phone calls), videoconference capability (one-to-one, group meetings), screen share capability (ability to share your screen with different documents), healthcare-based (previous use in medicine), pricing, supported operative systems and platforms, communications protection (encryption), extra security layer, security measures in group meetings (administration of pass-invitations), Security Standard Compliance, and Privacy policy.

## Results

Twenty-six videoconferencing software programs were identified (Tables 1, 2; Supplementary Figure 1). Regarding the technical capabilities, 13/26 (50.0%) were designed specifically for use in healthcare. All requested information was only found in 5/26 (19.2%) applications, including frequently asked information by users such as pricing in 11/26 (42%), and security information in 11/26 (43%) with 6 out 26 (23.0%) were both compliant with HIPAA and GDPR. All detailed information and definitions are included in Tables 1, 2.

**Table 1:** Widely Known applications for videoconferencing.

	**General features**					**Specific features**				**Security features**				
**Application**	**Chat**	**Calls**	**Video Calls**	**File Sharing**	**Group video-meetings**	**Screen share**	**Healthcare based**	**Pricing(free/license)**	**Supported OS and platforms**	**Communications protection**	**Extra security layer**	**Security in group meetings**	**Security Standards Compliance**	**Privacy Policy Statement**
Facebook messenger	✓	✓	✓	✓	✓ Up to 50 users	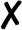	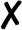	Free	Windows, MacOs, iOS, Android	E2EE	2-step verification (2FA)	Invitation, Admin control (Messenger Rooms)	SOC2, GDPR	https://www.facebook.com/about/privacy
FaceTime	✓	✓	✓	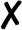	✓ Up to 32 users	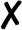	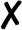	Free	MacOs. iOs	E2EE	2FA, Face ID and iPhone security	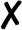	?	https://support.apple.com/en-us/HT209110
Google Duo	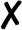	✓	✓	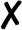	✓ Up to 12 users	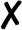	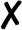	Free	Movil based: Android, iOs, Web browser-based	E2EE	2FA, Google Account security	Invitation and user block option	HIPAA - BAA, GDPR	https://policies.google.com/privacy
Google Hangouts (aka Meet or Workspace)	✓	✓	✓	✓	✓ Up to 10 users	✓	✓	Contact Sales	Android, iOs, Web browser-based	IETF, SRTP and DTLS client-Server	2FA Advanced protection program (APP) SSO and Google's MFA	Invitation, admin control PIN	HIPAA HITRUST SOC2 GDPR	https://policies.google.com/privacy
Jitsi Meet	✓	✓	✓	✓	✓ Without limit	✓	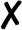	Free and License	Windows, Linux, MacOs, iOS, Android	E2EE DTLS-SRTP	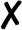	Password Admin control (every user is a moderator)	?	https://jitsi.org/meet-jit-si-privacy/
Line	✓	✓	✓	✓	✓ Up to 200 users	✓	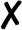 No (only on Geater Tokyo Area)	Free	Windows, MacOs, iOS, Android	E2EE	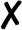	Invitation	?	https://help.line.me/line/android/pc?lang=en
Signal	✓	✓	✓	✓	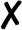 Up to 8 and no limits with chat	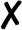	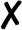	Free	Windows, Linux, MacOs, iOS, Android	E2EE	Screen Lock	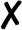	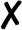	https://signal.org/legal/#privacy-policy
Skype for business (part of Office 365; formerly Microsoft Lync)	✓	✓	✓	✓	✓ Up to 50 users	✓	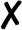	License	Windows, Linux, MacOs, iOS, Android	EE2E (private conversation)	2FA	Invitation, Admin control	GDPR, HIPAA, HITRUST, HITECH, CCPA.	https://privacy.microsoft.com/es-ES/privacystatement
Telegram	✓	✓	✓	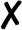	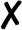 Up to 1000 users and no limits in chat	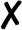	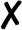	Free	Windows, Linux, MacOs, iOS, Android	E2EE (secret chat)	2FA, block code, secret chats, and active sessions	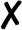	GDPR	https://telegram.org/privacy
WeChat	✓	✓	✓	✓	✓ Up to 9 users and 500 in chat	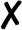	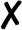	Free and License	Windows, Web browser-based, MacOs, iOS, Android	TLS client-Server	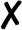	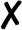	EEA	https://www.wechat.com/en/privacy_policy.html
WhatsApp	✓	✓	✓	✓	✓ Up to 8 users and 256 in chat	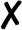	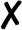	Free	Windows, Web browser-based, MacOs, iOS, Android	E2EE	2FA	Invitation	GDPR, EEA	https://www.whatsapp.com/legal/updates/privacy-policy-eea/?lang=en
Zoom	✓	✓	?	✓	✓ Up to 100 users on the paid version	✓	✓	License	Windows, Linux, MacOs, iOS, Android	E2EE, DTLS	2FA, SSO	Invitation, Password, Admin control	HIPAA - BAA, PHIPA/PIPEDA, SOC2	https://zoom.us/en-us/privacy.html
Teams	✓	✓	✓	✓	✓ Up to 20 users and 250 in chat	✓	✓	License	Windows, Linux, MacOs, iOS, Android, Web browser-based	E2EE	2FA	Invitation, Password, Admin control	HIPAA, HITECH, SOC2, HITRUST, GDPR	https://privacy.microsoft.com/en-gb/privacystatement

*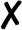, Feature not available; ?, Missing feature; ✓, Information is available. This table was designed and created by the Universidad de Burgos and Hospital Universitario de Burgos. The features of the apps and their security and privacy details shown in the table are based on the available information on April 12, (2021). If an application has two versions of its product and one of them is healthcare-based, only the healthcare-based was analyzed. The features of each health-based platform were gathered for the complete version (e.g., If there are three pricing plans for an application, the features of the complete one were selected). ? in any column means that we have not found any information. HIPAA and SOC2 (and others) are additional security standards. ADFS, Active Directory Federation Services (AD FS). It is a software developed by Microsoft. Provide users with unique credentials to access all applications within the same organization. AES, Advanced Encryption Standard is a specification for the encryption of electronic data. It was established by the U.S. National Institute of Standards and Technology (NIST) in 2001. AES can use three different key lengths: 128, 192, and 256. APP, Advance Protection Program. It is a system developed by Google, protecting users from all kinds of intentional online attacks. New protections are added automatically to deal with emerging threats. BAA, Business associate agreement. BAAs are hybrid contractual and regulatory instruments, meaning they both satisfy HIPAA regulatory requirements and create liability between the parties. CCPA, California Consumer Privacy Act (CCPA). The CCPA, approved in 2018, gives consumers more control over businesses' personal information about them. The CCPA regulations also guide how to implement the law. CSF, Common Security Framework. It is a set of documented policies and controls that govern an organization's security implementation and ongoing management. COPPA, Children's Online Privacy Protection Rule. It is a privacy act that imposes specific requirements on operators of websites or online services that collect personal information from children under 13 years of age. DTLS, Datagram Transport Layer Security. It is a protocol that provides privacy in communications. This protocol secures the client/server applications to avoid unwanted eavesdropping, unauthorized access, or message modification. E2EE, End-to-end encryption. It is a communication system where only the end users can read the messages. No third party can decrypt the data that is being communicated or stored. ECDHE-RSA, The acronym RSA comes from the surnames of Ron Rivest, Adi Shamir, and Leonard Adleman, who publicly described the algorithm in 1977. RSA is a public-key cryptosystem that is widely used for secure data transmission. ECDHE (Elliptic Curve Diffie-Hellman) is an anonymous key establishment protocol. EEA, European Economic Area. Face-ID, A facial recognition system that allows biometric authentication, it was designed and developed by Apple. GDPR, General Data Protection Regulation. The European regulation on the protection of personal data. Effective since May 24, 2016. H.235, The protocol used to authenticate trusted H.323 endpoints and encrypt the media stream during meetings. H.323 is a recommendation from the ITU Telecommunication Standardization Sector. HIPAA, Health Insurance Portability and Accountability Act of 1996. A USA Act was created primarily to modernize the flow of healthcare information. Includes how the healthcare and healthcare insurance industries should maintain personal information to avoid frauds and thefts. HITECH, Health Information Technology for Economic and Clinical Health Act. Enacted in 2009, it promotes and expands the adoption of health information technology. HITRUST, Prescriptive set of controls that meet the requirements of multiple regulations and standards, for example, HIPAA. Hardware as a Service program, It is a cloud computing model in which it is possible to pay for hardware resources without worrying about buying hardware or keeping the products updated. IETF, Internet Engineering Task Force. It is an organization that promotes the development of open standards, particularly for communications through the Internet. ISO, International Organization for Standardization. Develop and publish International Standards. PHIPA, Personal Health Information Protection Act. The legislation was established in November 2004. It is one of two components of the Health Information Protection Act (HIPAA). PIN, Personal Identification Number. Also called PIN code is a numeric/alpha-numeric passcode used to authenticate a user accessing a system. PIPEDA, Personal Information Protection and Electronic Documents Act. It is a Canadian law approved in 2000 to promote consumer trust in electronic commerce and protect personal information. SAML, Security Assertion Markup Language. SAML is an open standard based on the XML-based markup language; it is used to exchange authentication and authorization data between parties, particularly between an identity provider and a service provider. SaaS, Software as a Service. It is a cloud computing model in which it is possible to pay for the use of a particular software without worrying about buying or operating that software. SIP, Session Initiation Protocol. It is a protocol used for initiating, maintaining, and terminating real-time sessions that includes voice, video, and messaging applications. It is used for private voice and video calls. SHA, Secure Hash Algorithms. They are a family of cryptographic hash functions published by the National Institute of Standards and Technology (NIST). SHA is used as a checksum to verify data integrity. SCEP, Simple Certificate Enrollment Protocol. Does IETF create a protocol designed to make the request and issuing of digital certificates as simple as possible. SOC2, System and Organization Controls, there are defined three levels SOC1, SOC2, and SOC3. It is an audit that measures the effectiveness of a cloud system based on the Principles and Criteria of the American Institute of Certified Public Accountants (AICPA). SSO, Single Sign-On. An authentication scheme allows users to log in with a single ID and password to several related yet independent software systems. SRTP-RTP, Secure Real-time Transport Protocol. An extension of the Real-Time Transport Protocol adds security features, such as message authentication, confidentiality, and response protection, mainly intended for VoIP communications. TLS-SSL, Transport Layer Security and its now-deprecated predecessor, Secure Sockets Layer. They are protocols for web browsers and servers that allow the authentication, encryption, and decryption of data sent over the Internet. MFA/2FA, Multi-factor authentication (MFA) or two-factor authentication (2FA). It is a method that reinforces the security of the applications, granting access to the system only after a user presents two or more different proofs of their identity*.

**Table 2:** Specific healthcare based applications.

	**General features**					**Specific features**				**Security features**				
**App**.	**Chat**	**Call**	**Video Calls**	**File Share**	**Group video-meeting**	**Screen share**	**Health-care based**	**Pricing(Free/License)**	**Supported OS and platforms**	**Communications protection**	**Extra security layer**	**Security in group meetings**	**Security Standards Compliance**	**Privacy Policy Statement**
Coviu	✓	✓	✓	✓	✓ up to 6 users in-clinic license	✓	✓	License	Web browser-based	E2EE, TLS 1.2, ECDHE_RSA with P-256 and AES_128. Coviu call, data, video and audio: DTLS-SRTP	Azure SSO, ADFS SSO, Firewall and proxy settings, API	Invitation, Waiting Area, Meeting administrator management, Security groups	HIPAA	https://www.coviu.com/en-au/privacy
Doxy.me	✓	✓	✓	✓	✓ Up to 10 users	✓ from Professional edition onwards	✓	Free and License	Web browser-based	E2EE, TLS, AES 128 with SHA256	SSO	Room passcode, access control Invitation to meeting through email	HIPAA-BAA, PHIPA/PIPEDA, HITECH, GDPR	https://doxy.me/en/privacy-policy/
Thera platform	✓	✓	✓	✓	✓ Number of users not specified.	✓ from Pro edition onwards	?	License	Web browser-based	Video encryption. Website encryption 2048-bit SSL 256-bit. Data transfer encryption. Encrypted database backups. Server encrypted AES-256 algorithm	SS0 (from Pro edition onwards)	Waiting room	HIPAA-BAA	https://www.theraplatform.com/about/privacy
Poly (ZOOM based app)	✓	✓	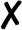	✓	✓ up to 100 users and 100 on the paid version	✓	✓	Zoom Hardware-as-a-Service program	Windows, Mac, Android, iOS	AES-256 encryption Simple Certificate Enrollment Protocol	Remote logging with support for TLS and local account. Also, login port lockout	Authenticated access to admin menus, web interface and APIs, and security profiles	GDPR, EEA, HIPPA	https://www.poly.com/us/en/legal/privacy/privacy-policy
Meetrix.io (Jitsi based)	✓	✓	✓	✓	✓ up to 500 users	✓	✓	Contact Sales	Web browser-based	E2EE DTLS-SRTP	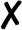	Password, Admin control:every user is a moderator	HIPAA	https://www.vidyohealth.com/privacy-policy
Vidyo Health	✓	✓	✓	?	✓ Number of users not specified.	✓	✓	Contact Sales	Windows, MacOs, iOS, Android, Web browser-based	TLS, SRTP, H.235, and AES 128-bit encryption	?	?	HIPAA	https://www.vidyohealth.com/privacy-policy
V2MD by Medisprout	✓	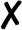	✓	✓	✓ Number of users not specified.	?	?	License	Apple iOS, Android	Secure Socket Layer technology	?	?	HIPAA	?
Cisco Jabber	✓	✓	✓	✓	✓ Up to 600 users	✓	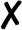	Free and License	Windows, Mac, Android, iOS	?	?	Invitation	?	https://www.cisco.com/c/en/us/about/legal/privacy-full.html
Univago	?	?	✓	✓	✓ up to 30	✓ from Professional edition onwards	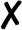	Contact Sales	Web browser based	SSL/TLS, DTLS, SRTP/AES, SSL/TLS	?	Unique Meeting ID, PIN codes, encryption, lock meetings	HIPAA	https://www.yorktel.com/privacy-policy/
Medweb	✓	?	✓	?	?	?	✓	Contact Sales	Windows, Android, iOS	Secure Socket Layer	?	?	HIPAA	https://www.medweb.com/medweb-software-privacy-policy
Teladoc.Health	?	?	✓	?	✓ Number of users not specified	?	✓	Contact Sales	SaaS: web, desktop, mobile devices	?	SSO user access control	?	HITRUST CSF HIPAA AdvaMed Certified	https://teladochealth.com/privacy-policy/
GlobalMed (eNcounter®)	?	?	✓	?	✓ Number of users not specified	?	✓	Contact Sales	?	Secure Socket Layer	?	?	HIPAA, ISO, FICSMA, HITRUST	https://www.globalmed.com/legal/privacy-statement/
SBR Health/Vidyo partner	?	?	?	?	?	?	✓	Contact Sales	?	?	?	?	HIPAA Children Online Privacy Protection Act	https://www.sbrhealth.com/privacy

*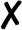, Feature not available; ?, Missing feature; ✓, Information is available. This table was designed and created by the Universidad de Burgos and Hospital Universitario de Burgos. The features of the apps and their security and privacy details shown in the table are based on the available information on April 12, (2021). If an application has two versions of its product and one of them is healthcare-based, only the healthcare-based was analyzed. The features of each health-based platform were gathered for the complete version (e.g., If there are three pricing plans for an application, the features of the complete one were selected). ? in any column means that we have not found any information. HIPAA and SOC2 (and others) are additional security standards. ADFS, Active Directory Federation Services (AD FS). It is software developed by Microsoft. Provide users with unique credentials to access all applications within the same organization. AES, Advanced Encryption Standard is a specification for the encryption of electronic data. It was established by the U.S. National Institute of Standards and Technology (NIST) in 2001. AES can use three different key lengths: 128, 192, and 256. APP, Advance Protection Program. It is a system developed by Google, protecting users from all kinds of intentional online attacks. New protections are added automatically to deal with emerging threats. BAA, Business associate agreement. BAAs are hybrid contractual and regulatory instruments, meaning they both satisfy HIPAA regulatory requirements and create liability between the parties. CCPA, California Consumer Privacy Act (CCPA). The CCPA, approved in 2018, gives consumers more control over businesses' personal information about them. The CCPA regulations also guide how to implement the law. CSF, Common Security Framework. It is a set of documented policies and controls that govern an organization's security implementation and ongoing management. COPPA, Children's Online Privacy Protection Rule. It is a privacy act that imposes specific requirements on operators of websites or online services that collect personal information from children under 13 years of age. DTLS, Datagram Transport Layer Security. It is a protocol that provides privacy in communications. This protocol secures the client/server applications to avoid unwanted eavesdropping, unauthorized access, or message modification. E2EE, End-to-end encryption. It is a communication system where only the end users can read the messages. No third party can decrypt the data that is being communicated or stored. ECDHE-RSA, The acronym RSA comes from the surnames of Ron Rivest, Adi Shamir, and Leonard Adleman, who publicly described the algorithm in 1977. RSA is a public-key cryptosystem that is widely used for secure data transmission. ECDHE (Elliptic Curve Diffie-Hellman) is an anonymous key establishment protocol. EEA, European Economic Area. Face-ID, A facial recognition system that allows biometric authentication, it was designed and developed by Apple. GDPR, General Data Protection Regulation. The European regulation on the protection of personal data. Effective since May 24, 2016. H.235, It is the protocol used to authenticate trusted H.323 endpoints and encrypt the media stream during meetings. H.323 is a recommendation from the ITU Telecommunication Standardization Sector. HIPAA, Health Insurance Portability and Accountability Act of 1996. A USA Act was created primarily to modernize the flow of healthcare information. Includes how the healthcare and healthcare insurance industries should maintain personal information to avoid frauds and thefts. HITECH, Health Information Technology for Economic and Clinical Health Act. Enacted in 2009, it promotes and expands the adoption of health information technology. HITRUST, Prescriptive set of controls that meet the requirements of multiple regulations and standards, for example, HIPAA. Hardware as a Service program. It is a cloud computing model in which it is possible to pay for hardware resources without worrying about buying hardware or keeping the products updated. IETF, Internet Engineering Task Force. It is an organization that promotes the development of open standards, particularly for communications through the Internet. ISO, International Organization for Standardization. Develop and publish International Standards. PHIPA, Personal Health Information Protection Act. The legislation was established in November 2004. It is one of two components of the Health Information Protection Act (HIPAA). PIN, Personal Identification Number. Also called PIN code is a numeric/alpha-numeric passcode used to authenticate a user accessing a system. PIPEDA, Personal Information Protection and Electronic Documents Act. It is a Canadian law approved in 2000 to promote consumer trust in electronic commerce and protect personal information. SAML, Security Assertion Markup Language. SAML is an open standard based on the XML-based markup language; it is used to exchange authentication and authorization data between parties, particularly between an identity provider and a service provider. SaaS, Software as a Service. It is a cloud computing model in which it is possible to pay for the use of a particular software without worrying about buying or operating that software. SIP, Session Initiation Protocol. It is a protocol used for initiating, maintaining, and terminating real-time sessions that includes voice, video, and messaging applications. It is used for private voice and video calls. SHA, Secure Hash Algorithms. They are a family of cryptographic hash functions published by the National Institute of Standards and Technology (NIST). SHA is used as a checksum to verify data integrity. SCEP, Simple Certificate Enrollment Protocol. Does IETF create a protocol designed to make the request and issuing of digital certificates as simple as possible. SOC2, System and Organization Controls, there are defined three levels SOC1, SOC2, and SOC3. It is an audit that measures the effectiveness of a cloud system based on the Principles and Criteria of the American Institute of Certified Public Accountants (AICPA). SSO, Single Sign-On. An authentication scheme allows a user to log in with a single ID and password to several related yet independent software systems. SRTP-RTP, Secure Real-time Transport Protocol. An extension of the Real-Time Transport Protocol adds security features, such as message authentication, confidentiality, and response protection, mainly intended for VoIP communications. TLS-SSL, Transport Layer Security and its now-deprecated predecessor, Secure Sockets Layer. They are protocols for web browsers and servers that allow the authentication, encryption, and decryption of data sent over the Internet. MFA/2FA, Multi-factor authentication (MFA) or two-factor authentication (2FA). It is a method that reinforces the security of the applications, granting access to the system only after a user presents two or more different proofs of their identity*.

## Discussion

This article summarizes the main technical and security aspects of commercially available videoconferencing software for healthcare use, features that a clinician should consider while choosing a videoconferencing software. Overall, the main features of current videoconferencing software are applicable to healthcare in general and they are not specific to movement disorders. Surprisingly, we collected complete data regarding capability and security in less than 20% of videoconferencing software platforms in use, suggesting that information about technical capabilities and data security is not easily and openly accessible for interested future users. In addition, complete and explicit information on whether the vendor/subcontractors have access to the data, including the video and other medical information, was also not entirely available for review.

In this review, we have not included other essential aspects for a successful videoconference visit. Firstly, the size of the room and the number of participants where the videoconference is conducted. These aspects will determine the exact type of equipment (camera, microphone, speakers, etc.) we will need to get good video and audio quality. Secondly, it is recommended to use videoconference etiquette tips, including adequate lighting in a professional environment, eliminating background noise and looking straight at the camera, dressing professionally, and avoiding multitasking^1^ (9).

Given the significantly increased use of remote care delivery during the Covid-19 pandemic, neurologists are facing an opportune time to expand the access to patients with movement disorders using videoconferencing tools (3, 10). A shift to video conferencing visits must be accompanied by efforts to prepare for and protect against breaches of security and privacy. Concern over such breaches is one of the many barriers and challenges against the more widespread adoption of telemedicine (2). Cybersecurity must be appropriately addressed to continue providing the best and safest care to our patients. To date, the most common strategies to enhance the cybersecurity of videoconferences include (1) password requirements, preventing unsolicited visitors from joining the meeting; (2) careful selection of software with the involvement of the IT department; (3) downloading the official release with regular updates for security patches; (4) ensuring there is no storage of video or medical data by the vendor; (5) identifying and monitoring attendees, with an alert when new attendees join the videoconference; (6) setting up waiting rooms that allow the organizer to determine whether those waiting are eligible to participate; and (7) encrypting meeting recording, making the information unreadable when obtained by third parties.

Presently and in the future, telemedicine may continue to be necessary to overcome infectious or other public health disasters/pandemics, where a healthcare response can be mobilized in a short period of time (5). In response to Covid-19 pandemic, telephone calls, messaging apps, or video visits have replaced or supplemented outpatient clinics (5). New regulations for telemedicine were created, and for example, in South Korea, the illegal status was lifted to follow established patients through telemedicine (5). Governments from several countries have initiated legislation to promote and regulate telemedicine and/or amended their prior restrictive regulations, including the US^2^, Europe (11), and Saudi Arabia ^3^.

The strength of our conclusions is tempered by some limitations, including selection bias given the lack of information on non-English-based videoconference software. There are also important aspects to users which were not included in our table, such as “How” to conduct a videoconference (with a laptop, mobile phones, tablets) and with “Whom” (with patients, caregivers, or other health professionals), which are decisive critical factors for a successful videoconference in certain populations. We also did not elaborate on the ongoing debate concerning the best indications for the use of videconference visits in movement disorders. However, most would appear to agree that videoconferencing should be reserved for follow-up visits, intermingled with in-person visits to the hospital whenever possible, but preferably not for making a diagnosis in a new patient (12, 13). Previous literature has shown a digital gap and poor eHealth literacy (14), especially in elderly, uneducated patients, limiting telemedicine's usefulness in certain groups of patients. An extra layer of support is sometimes required to facilitate and expand the use of videoconferences by patients, including caregivers' assistance, telemedicine health personnel assistants (“telepresenters”), and the use of health care facilities designed to establish videoconferences. One of the most established telemedicine programs to date is “The Ontario Telemedicine Network” (OTN) in Canada, which employs strategies to ensure that even patients with limited technological capabilities can access telemedicine care. The OTN supports all practice specialties, including movement disorders and those with deep brain stimulation (DBS)^4^. Therefore, an optimal telemedicine program with videoconferencing should balance security aspects with user-friendliness for patients and providers, cost, browser integration, operating systems, mobile platforms, and electronic health record integrations.

In conclusion, we have described the main technical and security features of the most popular videoconferencing tools used at present. Our data serves as a checklist guide for practitioners to understand what features should be examined when choosing a videoconference software and available options. However, because technology is a science characterized by a fast evolution, it is necessary to keep updating this type of information to neurologists interested in developing telemedicine programs.

## Author Contributions

All authors confirm that they have significantly contributed to the review of the literature, writing and review of this article.

## Funding

This work was supported by the project PI19/00670 of the Ministerio de Ciencia, Innovación y Universidades, Instituto de Salud Carlos II, Spain.

## Conflict of Interest

The authors declare that the research was conducted in the absence of any commercial or financial relationships that could be construed as a potential conflict of interest.

## Publisher's Note

All claims expressed in this article are solely those of the authors and do not necessarily represent those of their affiliated organizations, or those of the publisher, the editors and the reviewers. Any product that may be evaluated in this article, or claim that may be made by its manufacturer, is not guaranteed or endorsed by the publisher.

## Footnotes

^1^Available online at: https://www.vault.com/blogs/workplace-issues/best-practices-for-video-conference-etiquette.

^2^Available online at: https://www.commonwealthfund.org/publications/issue-briefs/2021/jun/states-actions-expand-telemedicine-access-covid-19.

^3^Available online at: https://nhic.gov.sa/en/Initiatives/Documents/TheGoverningRulesOfTelehealthEnglishEstablishingRules.pdf.

^4^Available online at: https://otn.ca; Visited at May, 10th 2021.
