# Supplementary material for: Videoconferencing Software Options for Telemedicine: A Review for Movement Disorder Neurologists
Source: Front Neurol. 2021 Oct 11;12:745917. doi: 10.3389/fneur.2021.745917 (PMC8542984; doi:10.3389/fneur.2021.745917)
Supplement: Supplementary file 1 [file Data_Sheet_1.PDF]

Supplementary figure 1

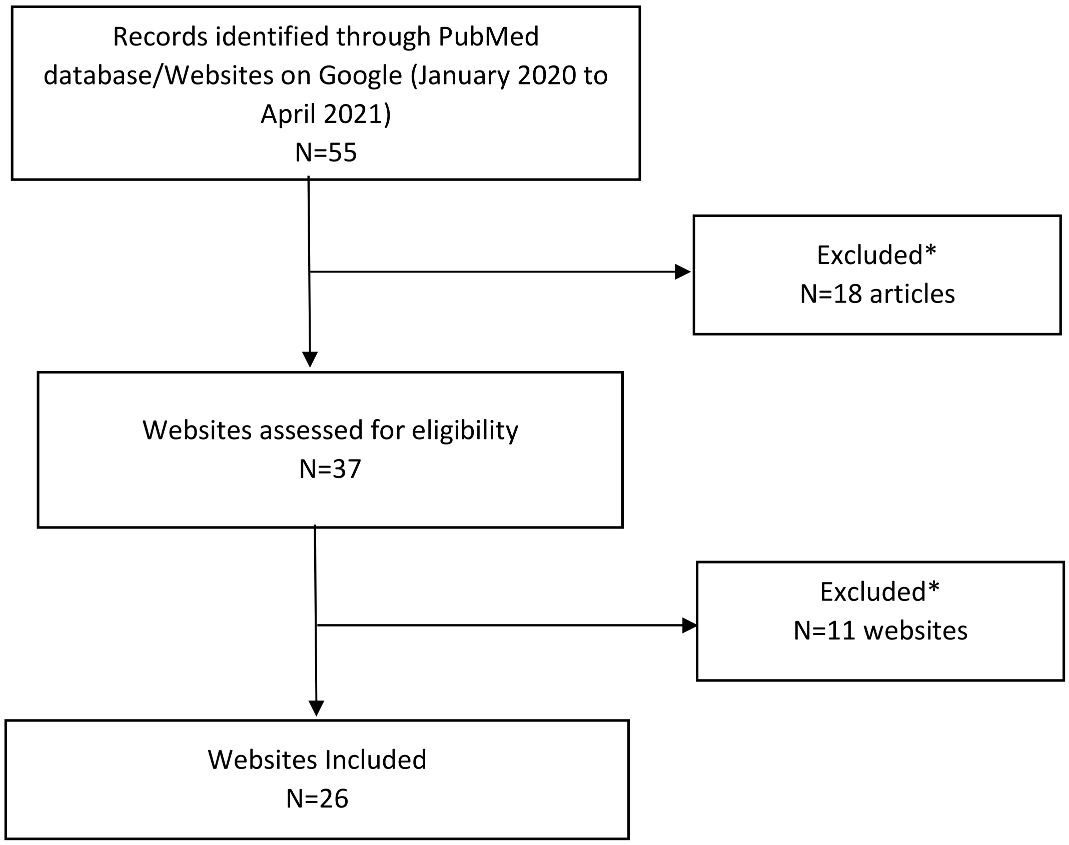

Figure 1-Prisma flow diagram for identification of relevant studies/websites. *Reasons for exclusion: non-matched inclusion criteria.
